# Rapid response in the COVID-19 pandemic: a Delphi study from the European Pediatric Dialysis Working Group

**DOI:** 10.1007/s00467-020-04584-6

**Published:** 2020-05-17

**Authors:** Fabian Eibensteiner, Valentin Ritschl, Gema Ariceta, Augustina Jankauskiene, Günter Klaus, Fabio Paglialonga, Alberto Edefonti, Bruno Ranchin, Claus Peter Schmitt, Rukshana Shroff, Constantinos J. Stefanidis, Johan Vande Walle, Enrico Verrina, Karel Vondrak, Aleksandra Zurowska, Tanja Stamm, Christoph Aufricht, On behalf of the European Pediatric Dialysis Working Group

**Affiliations:** 1 Division of Pediatric Nephrology and Gastroenterology, Comprehensive Center for Pediatrics, Medical University of Vienna, Waehringer Guertel 18-20, 1090 Vienna, Austria; 2 Section for Outcomes Research, Center for Medical Statistics, Informatics, and Intelligent Systems, Medical University of Vienna, Vienna, Austria; 3 Department of Pediatric Nephrology, University Hospital Vall d’ Hebron, Barcelona, Spain; 4 Pediatric Center, Institute of Clinical Medicine, Vilnius University, Vilnius, Lithuania; 5 Department of Pediatric Nephrology, KfH Children’s Kidney Center, Marburg, Germany; 6 Pediatric Nephrology, Dialysis and Transplant Unit, Fondazione IRCCS Ca’ Granda Ospedale Maggiore Policlinico, Milan, Italy; 7 Department of Pediatric Nephrology, Hôpital Femme Mère Enfant, Hospices Civils de Lyon, Lyon, France; 8 Pediatric Nephrology, Center for Child and Adolescent Medicine, Heidelberg, Germany; 9 Renal Unit, UCL Great Ormond Street Hospital for Children NHS Foundation Trust, and Institute of Child Health, London, UK; 10 Department of Pediatric Nephrology, Mitera Children’s Hospital, Athens, Greece; 11 Department of Pediatric Nephrology, Utoped, Universitair Ziekenhuis Gent, Ghent, Belgium; 12 Dialysis Unit, Department of Pediatrics, IRCCS Giannina Gaslini, Genoa, Italy; 13 Department of Pediatric Nephrology, University Hospital Motol, Prague, Czech Republic; 14 Department of Pediatric Nephrology, Medical University of Gdansk, Gdansk, Poland

**Keywords:** COVID-19, Pandemic, Delphi, Dialysis, Transplantation

## Abstract

**Background:**

COVID-19 was declared a global health emergency. Since children are less than 1% of reported cases, there is limited information to develop evidence-based practice recommendations. The objective of this study was to rapidly gather expert knowledge and experience to guide the care of children with chronic kidney disease during the COVID-19 pandemic.

**Methods:**

A four-round multi-center Delphi exercise was conducted among 13 centers in 11 European countries of the European Pediatric Dialysis Working Group (EPDWG) between March, 16th and 20th 2020. Results were analyzed using a mixed methods qualitative approach and descriptive statistics.

**Results:**

Thirteen COVID-19 specific topics of particular need for guidance were identified. Main themes encompassed testing strategies and results (*n* = 4), changes in use of current therapeutics (*n* = 3), preventive measurements of transmission and management of COVID-19 (*n* = 3), and changes in standard clinical care (*n* = 3). Patterns of center-specific responses varied according to regulations and to availability of guidelines.

**Conclusions:**

As limited quantitative evidence is available in real time during the rapid spread of the COVID-19 pandemic, qualitative expert knowledge and experience represent the best evidence available. This Delphi exercise demonstrates that use of mixed methodologies embedded in an established network of experts allowed prompt analysis of pediatric nephrologists’ response to COVID-19 during this fast-emerging public health crisis. Such rapid sharing of knowledge and local practices is essential to timely and optimal guidance for medical management of specific patient groups in multi-country health care systems such as those of Europe and the US.

**Electronic supplementary material:**

The online version of this article (10.1007/s00467-020-04584-6) contains supplementary material, which is available to authorized users.

## Introduction

In December 2019, an outbreak of pneumonia of unknown etiology emerged in Wuhan, Hubei province, China. [1–3] The rapid worldwide spread of severe-acute-respiratory-syndrome-coronavirus-2 (SARS-CoV-2)-related disease (COVID-19) has led to its designation as a global health emergency. [2] By early March 2020, more than 100,000 infections and more than 4000 deaths worldwide were attributable to COVID-19. [4, 5] Most available information on COVID-19 currently stems from the Chinese experience, ultimately characterized by aggressive and uniform public health responses. [6, 7] In contrast, the federalized European states have not introduced uniform countermeasures, which may render their responses more comparable to the US than to the more centralized Chinese public health response. [6, 7]

As fewer than 1% of reported COVID-19 cases have been children, information about COVID-19 in the pediatric population is sparse. [5, 8–11] The majority of available information concerns the major risk populations, including adults with significant comorbidities and the elderly. [12–21] Consequently, there is an urgent need for information to guide the management of other patient populations. Development of such guidance is especially challenging in highly specialized fields with small target populations, such as pediatric nephrologists caring for children with kidney transplants, receiving dialysis, or being treated with immunosuppressive therapy for kidney disease. There is significant concern that such patients are more likely to develop severe disease due to SARS-CoV-2 infection; yet, they may have an increased risk of exposure due to the need to have ongoing clinical care, including dialysis and laboratory monitoring. The current pandemic provides a challenge to the international community to develop new approaches for rapid analysis and dissemination of information to guide the management of patients with unique healthcare needs.

We hypothesized that application of a mixed method qualitative approach among established international networks of expert groups could very rapidly assess relevant knowledge gaps and retrieve current and evolving evidence of clinical response patterns. [22, 23] To rapidly gather this expert knowledge for the care of children with kidney disease, dialysis, and kidney transplantation during the emerging COVID-19 pandemic in Europe, a Delphi exercise was conducted among the European Pediatric Dialysis Working Group (EPDWG).

## Methods

We conducted a four-round Delphi exercise among the Pediatric Nephrology experts from the European Pediatric Dialysis Working Group (EPDWG) using an email survey. The Delphi process provides structured communication, iteration with controlled feedback, and informed input, [24–26] thereby facilitating rapid synthesis of expert knowledge. The emerging COVID-19 pandemic is creating all over Europe a quickly changing and ever more challenging setting in which to provide pediatric nephrology care. This Delphi exercise was conducted within the 5 days from March, 16th, to March 20th, 2020, during the first week of statutory public curfews imposed by local governments across Europe in response to emerging hotspots of SARS-CoV-2.

### Participants

All active EPDWG members representing 16 centers from 13 European countries were asked to participate. At the time of initiation of this Delphi exercise, COVID-19 cases in varying numbers had been reported by local authorities in all EPDWG countries.

### Data collection and qualitative analysis

A Delphi exercise uses a series of questionnaires to collect data from multiple panelists. In contrast to other data-gathering techniques, Delphi studies use feedback processes (iterations), to develop a consensus concerning a specific topic. This allows participants to reassess their initial judgments at a later time based on information provided earlier. [24] The first Delphi round started with an open email invitation to all EPDWG members to share their current knowledge, experience, information, and guidance for COVID-19 prevention, diagnostics, and management in their target population. Replies were gathered for 24 h. Information provided by the EPDWG members was analyzed using a modified form of meaning condensation analysis. [27, 28] Email replies were first read through to gain an overview of the collected data, after which themes were extracted and clustered according to common meanings. Clustered themes and corresponding quotations for each center were synthesized in a summary of finding table sent to all participants. Thus, participants could see the findings for each theme and center, including their own. The goal of the second Delphi round was to edit the wording, propose amendments, and check correctness of the summaries extracted from the experts’ statements. A summary of findings containing the final version of clustered themes and participants’ quotations for each center was created. In the third Delphi round, participants were asked to complete information on existing themes they had not addressed previously. Replies from rounds 2 and 3 were collected within 48 h. After the third Delphi round, no new information emerged and data saturation was achieved. Modified meaning condensation analysis [27, 28] was further utilized to formulate thematic questions and accompanying answer statements to systematically categorize the whole range of the specific, gathered knowledge. A set of clustered thematic questions and answer statements was created (Table 1). For the fourth Delphi round, this thematic question and answer statement set was circulated, and experts were asked to rate each statement with “total agreement” or “no agreement” in accordance with their local practice. We did not aim at consensus due to the descriptive character of our Delphi exercise, but rather aimed for rapid sharing of knowledge and practices to allow improved guidance of local management.

**Table 1:** Summary of the 13 identified thematic questions, with corresponding frequencies for preformed answer statements among the 13 EPDWG centers

Thematic questions and answer statements	Number (%)
I. Which patients are tested for COVID-19?	
Symptomatic patients	13 (100)
Asymptomatic patients with kidney transplantation and epidemiologic risk	6 (46)
Asymptomatic patients with dialysis and epidemiologic risk	6 (46)
Asymptomatic patients with immunosuppression and epidemiologic risk	6 (46)
Asymptomatic patients with chronic disease and epidemiologic risk	5 (28)
Other asymptomatic patients with epidemiologic risk	3 (23)
Screening of asymptomatic patients without epidemiologic risk	0 (0)
II. Testing of Health Care Personnel for COVID-19?	
Screening of all asymptomatic staff members	1 (8)
Screening of asymptomatic staff members upon unprotected contact with suspected COVID-19 case	3 (23)
Screening of asymptomatic staff members upon unprotected contact with confirmed COVID-19 case	8 (62)
Screening of symptomatic staff members	9 (69)
Screening of symptomatic staff members with history of unprotected contact with suspected COVID-19 case	10 (77)
Screening of symptomatic staff members with history of unprotected contact with confirmed COVID-19 case	11 (85)
Sent home for quarantine and home office after possible contact	9 (69)
III. Patients with kidney transplantation and confirmed COVID-19?	1 (8)
IV. Patients with dialysis and confirmed COVID-19?	0 (0)
V. Continuation of immunosuppressive therapy?	13 (100)
VI. Discontinuation of ACE-I or ARB therapy?	0 (0)
VII. Discontinuation of Eculizumab therapy?	0 (0)
VIII. Dialysis ward triage system?	
Information to parents to call when child has COVID-19 symptoms	10 (77
Screening of patients upon entering the dialysis ward	7 (54)
Screening of patients upon entering the hospital	5 (38)
IX. Measures for prevention of SARS-CoV-2 transmission?	
Zero visitors or chaperons (including parents)	6 (46)
Only 1 chaperon allowed	10 (77)
Reduction of patient chaperons	10 (77)
Structural isolation via curtains, rooms, …	6 (46)
Laminar flow rooms	1 (8)
Separate transportation of patients to the dialysis center	5 (38)
Separation of physicians and nurses for each patient (with registry)	3 (23)
Spreading in different time slots with different teams to avoid coinfection	3 (23)
Face masks for patients	6 (46)
Face masks for physicians	8 (62)
Face masks and high protective gear (suits, face shields, …) for physicians	0 (0)
Face masks for nurses	9 (69)
Face masks and high protective gear (suits, face shields, …) for nurses	2 (15)
X. Preparations/provisions for dialysis of SARS-CoV-2-infected patients?	
Isolated rooms within own dialysis unit	8 (62)
Isolated rooms within pediatric hospital (e.g. PICU)	5 (38)
Isolated rooms at adult units	4 (31)
Isolated by separate time slots	4 (31)
Separation of medical staff (“COVID-19 teams”, physician and nurses)	5 (38)
XI. Changes of Pediatric Kidney Transplantation Program due to COVID-19?	
Discontinuation of living-related donor kidney transplantation	7 (54)
Discontinuation of deceased donor kidney transplantation	2 (15)
XII. Suspension of non-urgent care?	
Canceling of routine check ups	9 (69)
Canceling of elective procedures (e.g. elective surgery)	3 (23)
Suspension of routine visits of stable kidney transplant patients	3 (23)
Suspension of non-urgent appointments	7 (54)
XIII. Implementation of remote clinical work?	
Telephone calls with patients	12 (92)
Video calls with patients	4 (31)
E-Mails with patients	9 (69)
Telemonitoring of patients	5 (38)
Virtual online clinics for patients	4 (31)
No remote clinical work, but reduction of patients	4 (31)
Other, for example: home office with online tutoring and learning	5 (38)

*EPDWG* European Pediatric Dialysis Working Group, *COVID-19* SARS-CoV-2-related-disease, *SARS-CoV-2* severe-acute-respiratory-syndrome-coronavirus-2, *ACE-I* angiotensin-converting-enzyme inhibitors, *ARB* angiotensin II-receptor-blockers, *PICU* pediatric intensive-care unit

Descriptive statistics and appropriate graphs were utilized to analyze frequencies and differences in local preparation and practice of pediatric nephrologists for emerging COVID-19. The draft of the manuscript was sent to all participants for their comments, which were addressed in the revised manuscript. The manuscript was submitted after final approval by the EPDWG.

## Results

### Participants and response rates

The invitation to participate was sent out via email to 16 centers from 13 European countries, all active members of the EPDWG. Thirteen centers (81%) from 11 European countries of the EPDWG (85%) participated in this Delphi exercise. The participating centers were from Austria, Belgium, Czech Republic, France, Germany, Greece, Italy, Lithuania, Poland, Spain, and the United Kingdom. Each participant completed all four rounds of the Delphi process.

### Themes

During the first round of the Delphi exercise, 13 clustered themes were identified. All themes were included in the second and third rounds of the Delphi exercise. No new themes emerged after the second round, and data saturation was achieved. Among the 13 identified themes, two included testing for COVID-19, two included confirmed COVID-19 cases, three included changes in current therapies, three included preventive and preparatory measures of transmission and management of COVID-19, and three included changes in routine clinical care due to COVID-19.

### Statements of current practice

**Fig. 1:**
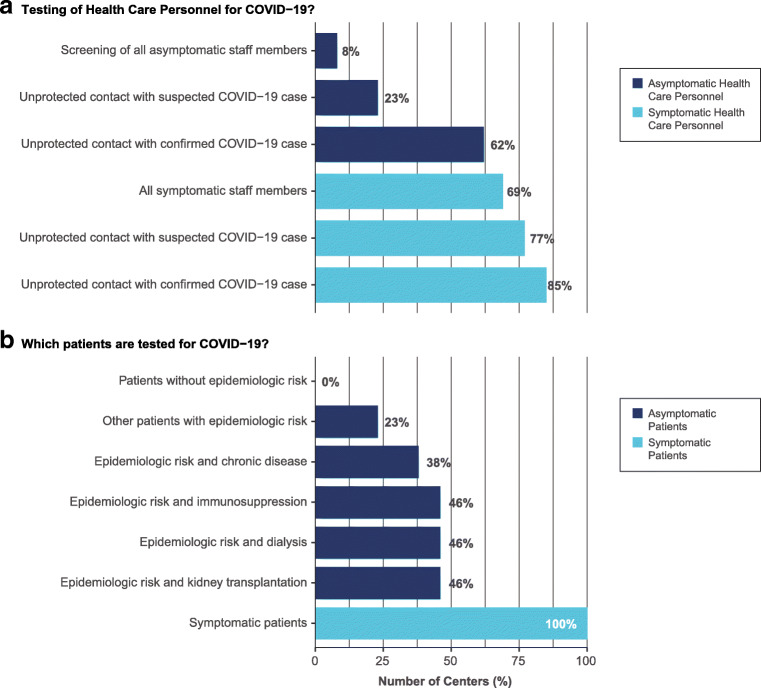
Local COVID-19 testing practices at the centers of the EPDWG

**Fig. 2:**
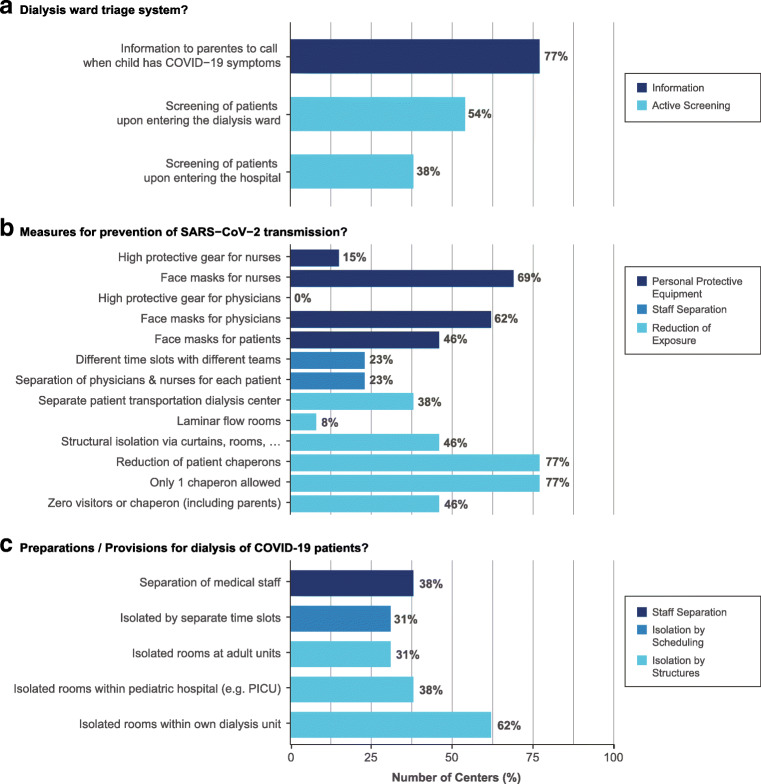
Preventive and preparatory measures of transmission and management of confirmed and suspected COVID-19 cases among EPDWG centers

**Fig. 3:**
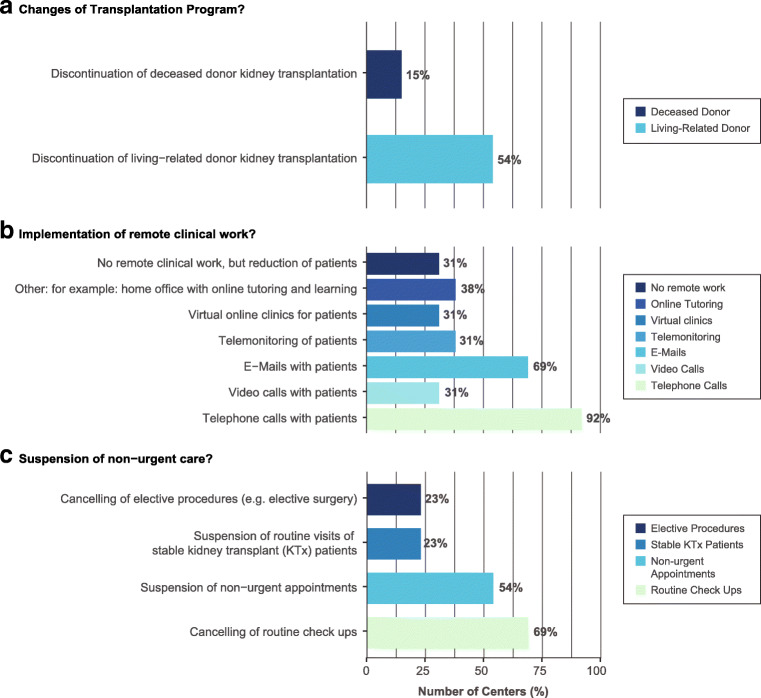
Changes in routine clinical care due to COVID-19 among EPDWG centers

For the final Delphi round, 13 thematic questions and a varying number of accompanying answer statements were formulated from the gathered data by use of modified meaning condensation analysis. [27, 28] Due to data saturation and clarity of statements from the first three Delphi rounds, only six thematic questions and their corresponding answer statements were included in the fourth round. Qualitative and quantitative results are, respectively, displayed in Table 1, Online Resource 1, and in Figs. 1, 2, and 3.

### Testing for COVID-19 at the centers of the EPDWG

Testing asymptomatic patients with increased risk (immunocompromised or dialysis patients) and epidemiologic risk was reported by 46% (6/13). Testing of patients with other or no chronic disease and epidemiologic risk was reported in 28% (5/13) and 23% (3/13), respectively. At the time of the study, no asymptomatic patients without epidemiologic risks were routinely tested in the centers of the EPDWG. Regular screening of all asymptomatic health care personnel (HCP) was performed in one center. Screening of asymptomatic HCP upon unprotected contact with suspected or confirmed COVID-19 cases was, respectively, performed in 23% (3/13) and 62% (8/13). Testing of symptomatic HCP for COVID-19 regardless of contact, after unprotected contact with suspected, or confirmed cases was conducted in 69% (9/13), 77% (10/13), and 85% (11/13), respectively. Home quarantine with home office after possible contact of HCP with COVID-19 cases is performed in 69% (9/13).

### Confirmed COVID-19 cases at EPDWG centers

Among 13 centers from 11 European countries, there have been no confirmed cases of COVID-19 in a pediatric dialysis patient and only one in a child with kidney transplant. The latter case occurred in Spain, with the second highest infection and mortality rate for COVID-19 among the 13 centers at the time. The two Italian centers reported no cases of SARS-CoV-2-positive children, despite Italy’s status as having the highest COVID-19 infection rate.

### Changes in current therapies among the EPDWG

All 13 centers of the EPDWG were in consensus for continuation of immunosuppressive therapy. Statements regarding prophylactic or post-COVID-19 diagnosis discontinuation of ACE-I or ARB therapy in children with renal disease all supported continuation of established therapies*.* Within the first round of our Delphi exercise, questions arose regarding discontinuation of Eculizumab maintenance therapy for complement-mediated kidney disease, such as atypical hemolytic uremic syndrome (aHUS). During the second and third Delphi rounds, this question was addressed, with consensus toward continuation of therapy.

### Preventive and preparatory measures of transmission and management of COVID-19

Seventy-seven percent (10/13) of EPDWG centers are providing recommendations to parents to contact their pediatric nephrology providers when their child has COVID-19-related symptoms. Thirty-eight percent (5/13) and 54% (7/13) of centers, respectively, perform active screening of patients upon entering the hospital or dialysis ward. Whereas some EPDWG dialysis units can provide isolated rooms for each patient and even laminar flow rooms, others rely on purely organizational measures. The Italian centers of the EPDWG issued guidance that all medical staff and sanitary professionals need to be organized in teams and efficiently protected to avoid co-infection. German centers implemented strategies to be prepared for upcoming delivery shortages, whereas the Czech Republic prescribed during the third Delphi round that face masks were obligatory in public sites. Frequencies for measures of prevention of SARS-CoV-2 transmission are given in Table 1. Isolation of cases in segregated structures within dialysis units, within the pediatric hospitals (e.g., Intensive Care Units) or within adult units was conducted in 62% (8/13), 38% (5/13), and 31% (4/13) of centers, respectively. Isolation by assignment of cases to different time slots was implemented in 31% of centers (4/13), and separation of medical staff was achieved in 38% (5/13).

### Changes in routine clinical care due to COVID-19

Fifty-four percent (7/13) of EPDWG centers suspended living-related donor (LRD) transplantation. Deceased donor (DD) kidney transplantation was discontinued in 15% of centers (2/13). At two centers, both LRD and DD transplantation were suspended during the last week of the Delphi exercise. Routine checkups and non-urgent appointments were canceled in 69% (9/13) and 54% (7/13) of centers, respectively. Routine visits for stable pediatric kidney transplant patients were suspended in 23% of centers (3/13). Cancelation of elective procedures and/or surgery was reported in 23% of centers (3/13). Figure 3 presents specific measures of remote clinical work implemented.

## Discussion

During an acute and rapidly evolving pandemic crisis such as the COVID-19 pandemic, it may appear impossible to generate evidence for recommendation of the management of specific patient groups within short periods of time using standard methodologies. Thus, current recommendations are mostly derived from the reported Chinese experience (and focus on high-incidence groups with high risk). [12–21] This lack of targeted clinical evidence will likely not change within highly specialized fields, such as all pediatric subspecialties. For example, in the recently published pediatric COVID-19 case series (almost exclusively based on Chinese data), no cases of children with kidney transplantation or on dialysis were reported. [5, 8–11]

In this context, expert knowledge, experience, and guidance may provide the best available “evidence”. Since COVID-19-specific reports of children with kidney disease, kidney transplantation, or dialysis were unavailable, as was guidance from respective professional societies, we conducted a Delphi exercise among experts in pediatric nephrology, representing 13 centers in 11 European countries (including two Italian centers). The urgency of the situation prompted us to conduct our Delphi exercise with a mixed method qualitative approach within 5 days, with four answer rounds of 24 h each.

Current advice for COVID-19 testing strategies relevant to the EPDWG depends on the stage of the outbreak in different areas, according to the European Centre for Disease Prevention and Control (ECDC). [29] Guidelines for COVID-19 testing of patients and HCP are issued by national authorities, but might be adapted by individual centers depending on local regulations and resources. [30, 31] For example, testing of symptomatic patients was uniformly performed in all centers as mandated by health authorities. However, testing of asymptomatic patients and asymptomatic HCP with varying risk factors markedly varied among EPDWG centers, likely reflecting decisions of individual expert teams responsible for clinical care, thus influenced by local expert attitudes and hospital policies. It is important to stress that criteria and strategies for COVID-19 testing are changing rapidly as the geographic spread of COVID-19 expands, as are physician attitudes and hospital policies. In accordance with current literature [5, 8–11], our study found no cases of confirmed COVID-19 in children with dialysis. However, we identified the first case of confirmed COVID-19 in a child with kidney transplantation in Spain. This child was doing well at the time of manuscript submission.

Guidance on management of immunosuppressive therapy in adult patients with kidney transplantation and COVID-19 has been recently issued. [32–36] Furthermore, arisen speculations on exacerbation of COVID-19 disease by concurrent treatment with ACE-I or ARB have been addressed by the European Society of Cardiology (ESC) and the American Heart Association (AHA), both strongly recommending continuation of these widely used drugs. [37, 38] Although no such guidance has been published for children, all 13 EPDWG centers were in complete consensus for continuation of these established therapies, while alert to appearance of additional data (for rapid dissemination across the network).

Strategies are emerging to counter acceleration of the COVID-19 pandemic in the face of shortages of resources and personal protective equipment. [31] Concerns about transmission of SARS-CoV-2 to HCP are also emerging more frequently. These might be even more relevant in the pediatric setting, as infected children appear to suffer fewer complications than do their adult HCP. Indeed, up to 20% of Italian HCP have been infected. [31] KDIGO has recently published guidelines, synthesized from the Chinese and Taiwanese Societies of Nephrology, that recommend separation of HCP and individual patients by location and time, in addition to entrance control, self-monitoring for symptoms, and use of appropriate protective equipment. [34] The EPDWG pattern of responses for prevention and management of SARS-CoV-2 demonstrated varying degrees of implementation of these recommendations across the 13 centers, primarily dependent on available resources, in addition to on local or regional governmental guidelines.

The COVID-19 pandemic led to suspensions of LRD and/or DD pediatric kidney transplantation programs in most EDPWG centers. These changes in transplantation policies were local decisions within the EPDWG centers and their hospitals, as no health authority regulations were available at the time of the study, and as European and US scientific societies recommended consideration of temporary suspension depending on local circumstances. [32, 39] Implementation of reduction in provision of non-urgent care similarly relied on local practices. Routine checkups and non-urgent appointments were being canceled in most EPDWG centers, whereas routine visits of stable kidney transplant patients and elective were suspended in about 25% of centers, resulting in significant reduction in direct patient contacts, as recently proposed by the Transplant Society in order to keep transplantation centers operational. [35] Among the EPDWG centers, remote clinical work measures implemented included phone- and video calls, online clinics, and telemonitoring for peritoneal dialysis patients.

Taken together, pediatric nephrology provider response patterns to the challenge of the COVID-19 pandemic have been diverse, as expectable from a multi-country European network in a federalized governmental environment. Responses concerning changes in current treatment were in consensus with recommendations from scientific bodies. However, state-of-the-art tends to be conservative, suggesting change to the status quo only upon presentation of clear evidence of the need for change. Some responses, such as those concerning testing strategies, prevention, and changes in routine care during the COVID-19 pandemic, varied widely among the EPDWG centers, reflecting in part rapid dynamic changes in the responses of national or local health authorities to escalation of the pandemic. Local hospital policies, physicians’ attitudes, and available resources also significantly influenced the diverse patterns of responses among EPDWG centers. Future studies, performing detailed longitudinal assessments of these interdependent variables will be needed to obtain a deeper understanding of determinants of individual response patterns. Such studies should be able to compare the efficacy of any country-specific responses to the COVID-19 pandemic.

The strengths of this Delphi exercise lie in the rapid response and communication of 13 expert centers across Europe, with consecutive qualitative data analysis in a thematic field for which no evidence and no guidance from international societies currently exist. The concise description of multi-country European response patterns may allow experts in other countries affected by this pandemic to base their own responses on an improved level of evidence for recommendations unavailable for the time being through standard methodologies. The COVID-19 pandemic is expanding in the United States with a lag of about 2 weeks compared to continental Europe. [4] This lag may provide crucial opportunities for US experts to learn from the European areas enduring a more advanced state of the pandemic.

Limitations of this study are inherent in the rapidly evolving pandemic and corresponding dynamics of changing regulations—thus the specific information on response patterns in this study is valid through the date of manuscript submission, and recommendations may change quickly thereafter. However, the consensus identification of specific COVID-19 topics of greatest relevance to a multi-country European expert group working in the midst of the pandemic should be generalizable and may facilitate development of relevant local guidance from other national expert groups or health authorities, supporting individual pediatric nephrology experts in their clinical decision making in a time of extreme uncertainty.

## Conclusion

In conclusion, this Delphi exercise exemplifies international cooperation and communication of experts during a rapidly emerging pandemic crisis. The use of a mixed method qualitative approach allowed retrieval of evidence on international clinical response patterns within an extremely short time. In times at which quantitative data and corresponding evidence under given circumstances is scarce, qualitative expert knowledge, experience, and guidance may be the best evidence available. These principles will apply to every situation in which quantitative evidence is lacking in the setting of an emerging international pandemic threat.

## Electronic supplementary material

ESM 1 (PDF 159 kb).

## Data Availability

No data or additional material is made available.

## Abbreviations

SARS-CoV-2: Severe-acute-respiratory-syndrome-coronavirus-2
COVID-19: SARS-CoV-2-related-disease
US: United States (of America)
EPDWG: European Pediatric Dialysis Working Group
HCP: Health care personnel
aHUS: atypical hemolytic uremic syndrome
LRD: living-related donor
DD: deceased donor
ECDC: European Centre for Disease Prevention and Control
ACE-I: Angiotensin-converting-enzyme inhibitors
ARB: Angiotensin II-receptor-blockers
ESC: European Society of Cardiology
AHA: American Heart Association
KDIGO: Kidney Disease: Improving Global Outcomes
HD: hemodialysis
ICU: intensive-care units
KTx: kidney transplantation
