# Supplementary material for: Rapid response in the COVID-19 pandemic: a Delphi study from the European Pediatric Dialysis Working Group
Source: Pediatr Nephrol. 2020 May 17;35(9):1669–78. doi: 10.1007/s00467-020-04584-6 (PMC7230035; doi:10.1007/s00467-020-04584-6)
Supplement: Supplementary file 1 — (PDF 159 kb). [file 467_2020_4584_MOESM1_ESM.pdf]

Rapid Response in the COVID-19 Pandemic: A Delphi Study from the European Pediatric Dialysis Working Group

Fabian Eibensteiner, MD[1], Valentin Ritschl, MSc[2], Gema Ariceta, MD, Prof.[3], Augustina Jankauskiene, MD, Prof.[4], Günter Klaus, MD, Prof.[5], Fabio Paglialonga, MD, Prof.[6], Alberto Edefonti, MD, Prof.[6], Bruno Ranchin, MD, Prof.[7], Claus Peter Schmitt, MD, Prof.[8], Rukshana Shroff, MD, Prof,[9], Constantinos J Stefanidis, MD, Prof.[10], Johan Vande Walle, MD, Prof.[11], Enrico Verrina, MD, Prof.[12], Karel Vondrak, MD, Prof.[13], Aleksandra Zurowska, MD, Prof.[14], Tanja Stamm, PhD, Prof.[2] and Christoph Aufricht, MD, Prof.[1] on behalf of the European Pediatric Dialysis Working Group

Affiliations:

[1]Division of Pediatric Nephrology and Gastroenterology, Comprehensive Center for Pediatrics, Medical University of Vienna, Vienna, Austria;

[2]Section for Outcomes Research, Center for Medical Statistics, Informatics, and Intelligent Systems, Medical University of Vienna, Vienna, Austria;

[3]Department of Pediatric Nephrology, University Hospital Vall d' Hebron, Barcelona, Spain;

[4]Pediatric Center, Institute of Clinical Medicine, Vilnius University, Vilnius, Lithuania;

[5]Department of Pediatric Nephrology, KfH Children's Kidney Center, Marburg, Germany;

[6]Pediatric Nephrology, Dialysis and Transplant Unit, Fondazione IRCCS Ca' Granda Ospedale Maggiore Policlinico, Milan, Italy;

[7]Department of Pediatric Nephrology, Hôpital Femme Mère Enfant, Hospices Civils de Lyon, Lyon, France;

[8]Pediatric Nephrology, Center for Child and Adolescent Medicine, Heidelberg, Germany;

[9]Renal Unit, UCL Great Ormond Street Hospital for Children NHS Foundation Trust, and Institute of Child Health, London, UK

[10] Department of Pediatric Nephrology, Mitera Children's Hospital, Athens, Greece.

[11]Department of Pediatric Nephrology, Utoped, Universitair Ziekenhuis Gent, Ghent, Belgium;

[12]Dialysis Unit, Department of Pediatrics, IRCCS Giannina Gaslini, Genoa, Italy;

[13]Department of Pediatric Nephrology, University Hospital Motol, Prague, Czech Republic;

[14]Department of Pediatric Nephrology, Medical University of Gdansk, Gdansk, Poland;

Address correspondence to:

Univ.-Prof. Dr. med. univ. Christoph Aufricht

Division of Pediatric Nephrology and Gastroenterology

Competence Center for Pediatrics

Medical University of Vienna

Waehringer Guertel 18-20

1090 Vienna, Austria

Phone: +43-1-40400-21115

christoph.aufricht@meduniwien.ac.at

Online Resource 1 Clustered thematic units with corresponding questions and typical responses among EPDWG centers

No.	Clustered thematic questions	Typical responses (Original quotations)
I	Which patients are tested for COVID-19?	*Only patients so ill that they have to be hospitalized are tested (because of lack of test facilities).*<br>*Patients with contact to, history of returning from COVID-19 risk areas or evidence of COVID-19 symptoms.*
II	Testing of Health Care Personnel for COVID-19?	*Asymptomatic staff with possible contacts can work with protection masks and should reduce contacts as much as possible.*<br>*If the professional is asymptomatic after exposure to a case, home isolation for 7 days, and working with a mask from 7-14 days is indicated.*<br>*As resources are so limited, if a member of staff has had contact with a confirmed case, but is asymptomatic, the test is not performed until there is a clinical reason to do so.*
III	Patients with kidney transplantation and confirmed COVID-19?	*We have a transplanted kid, doing fine.*
IV	Patients with dialysis and confirmed COVID-19?	*No dialyzed children have been infected.*
V	Continuation of immunosuppressive therapy?	*At the moment basing on the data obtained so far, we have no evidences to recommend any modification of the ongoing therapy.*<br>*We did not make any changes due the mild disease (the child was not treated with antiviral medication).*
VI	Discontinuation of ACE-I or ARB therapy?	*At the moment there is no strong evidence to recommend interrupting the use of the drug in all patients.*
VII	Discontinuation of Eculizumab therapy?	*Has anybody heard about an Eculizumab patient becoming infected? I have many questions from doctors and patients if to discontinue for a time further prophylactic Eculizumab infusions.*<br>*Regarding Eculizumab at the atypical Hemolytic Uremic Syndrome (aHUS) registry, we have been discussing it and by now the recommendation is to keep treatments active, as there is no evidence that Eculizumab changes the ability to fight COVID-19. We are trying to collect those data from now.*
VIII	Dialysis ward triage system?	*Information given to parents: phone call if fever or cough.*<br>*Triage when entering the hospital.*

No.	Clustered thematic questions	Typical responses (Original quotations)
		Patients and chaperones are initially evaluated by medical professionals. If their triage evaluation is negative, they can access the unit, whereas if there are any doubts regarding their clinical condition, they are sent for a mucosal swab test.
IX	Measures for prevention of SARS-CoV-2 transmission?	*Strict measures of home isolation as well as reduction of hospital admittance of patient and visitors, no parents in dialysis room, surgical mask for all nurses and doctors (we have not enough masks for children).*<br>*Sanitary professionals need to be efficiently protected and organized to work in teams to avoid simultaneous co-infection of operators.*<br>*Separate each doctor and nurse for each patient (and register it, to keep a track).*<br>*Mask and disinfectant saving strategies.*<br>*The Czech's government decision: masks for the whole population in public sites - streets, parks, public transportation, hospitals, opened shops etc. by nowadays midnight March 18th, 2020.*
X	Preparations / Provisions for dialysis of SARS-CoV-2 infected patients?	*Three separate routine hemodialysis (HD) rooms, two extracorporeal dialysis rooms (reverse isolation with under pressure) on the ward, two such HD rooms on the intensive-care units (ICU)".*<br>*Strict separation of medical personnel and patients to different rooms for apheresis and hemodialysis, with different time slots.*<br>*An isolated area in the adult unit, with a filter, where children on chronic hemodialysis who could possibly be infected can be sent with the support of one of our pediatric nurses.*<br>*Emergency HD will be done on the ICU*
XI	Changes of Pediatric Kidney Transplantation Program due to COVID-19?	*We would refrain from living related transplantation right now in view of uncertain risks and expectation of shortage of ICU capacities.*
XII	Suspension of non-urgent care?	*Cancelled most clinics (only urgencies), even for stable transplant kids, the idea is to keep people at home and far away from hospitals.*
XIII	Implementation of remote clinical work?	*Lots of phone calls with patients and families.*<br>*All possible clinics are performed online. Telemonitoring for peritoneal dialysis patients is provided.*

EPDWG = European Pediatric Dialysis Working Group
